# A review on prospects, challenges, stakeholders and strategies for the implementation of water, sanitation and hygiene in developing countries towards achieving Sustainable Development Goal 4

**DOI:** 10.4102/phcfm.v18i1.5376

**Published:** 2026-06-25

**Authors:** Esther Adekunle, Ferdinand Ottoh, Goddy Osimen, Adedotun O. Akinola

**Affiliations:** 1 Department of Political Science and International Relations, Covenant University, Ota, Nigeria; 2 Department of Political Science, University of Lagos, Akoka, Yaba, Nigeria; 3 Department of Architecture, Covenant University, Ota, Nigeria

**Keywords:** WASH, quality education, SDG 6, SDG 4, sustainable development, public health

## Abstract

Access to safe water, sanitation and hygiene (WASH) is a human right and a main driver of social well-being, economic progress and public health. This study presents a narrative review on the prospects, challenges, stakeholders and strategies regarding WASH implementation in developing countries. A literature search approach, fully dependent on the availability of data from secondary sources, has been adopted for the study. Improved sanitation and hygiene education from WASH boost health, learning and school attendance. However, equity, cost and maintenance are some of the barriers to WASH. Furthermore, success requires long-term investment, community engagement, collaboration across sectors, schools with infrastructure to support WASH, health education, monitoring of water quality and funding for sustainability mechanisms. This study contributes to the literature by revealing the prospects, challenges, stakeholders and strategies for the implementation of WASH in developing countries. This study will provide empirical data for researchers and policymakers towards achieving Sustainable Development Goal 4.

## Introduction

Access to safe water, sanitation and hygiene (WASH) is a human right and a main driver of social well-being, economic progress and public health.^1^ The United Nations set the Sustainable Development Goals (SDGs) in 2015, and among them, Goal 6 is to make sure that there is available and sustainable water and sanitation for all.^2^ But attainment of SDG 6 has an inherent interlinkage to other goals like SDG 4, covering quality education because better WASH conditions in schools enhance students’ health, school attendance and learning.^3^ Water sanitation and hygiene is one of many initiatives that developing countries’ governments have initiated in partnership with global institutions like the World Health Organization (WHO) and United Nations Children and Education Fund (UNICEF) aimed at improving the quality of education. The initiatives are formulated to support inclusive, enabling learning spaces with a common goal of improved enrolment, attendance and learning performance.^4,5^

Water, sanitation and hygiene reinforce UN SDG 6, providing access to safe water and sanitation. Water, sanitation and hygiene is a convergent notion that links water, sanitation and hygiene with improved health, education and general development outcomes.^5,6,7^ Apart from sanitation and access to clean water, WASH facilitates health, economic growth, gender equity and social development.^5^ The quality of education is determined by WASH through the provision of a safe, clean environment for learning.^8^ Safe drinking water gets rid of waterborne diseases, which decreases absenteeism. Safe water in schools ensures enrolment and attendance, at least where girls are concerned. For instance, a lack of access to a separate toilet is blamed for female absenteeism during menstruation. Better hygiene ensures a hygienic school environment, free from disease transmission.^9^ If communities are to be sustainable and resilient, WASH investment is a necessity. The impact it will have on education cannot be overestimated; hence, the realisation of WASH is a necessity to be attained as stated in SDG 6 and for its long-term returns to the students and society.

However, despite the outstanding accomplishments in the implementation of WASH across the globe, developing nations still struggle to overcome enormous challenges, including but not limited to poor infrastructure, a lack of funds and poor governance.^5^ In order for developing countries to meet these challenges, there is an urgent need for multi-stakeholder responses via governments, non-governmental organizations (NGOs), private sectors and local communities to contribute to developing appropriate policies and sustainable solutions.

Definitively, such interventions as capacity development, community mobilisation, innovative financing tools and embedding WASH into education systems are needed to facilitate progress towards meeting the SDGs.

This paper discusses prospects, challenges, stakeholders and implementation strategies for WASH in developing countries. The study presents an in-depth evaluation of the prevalent challenges and possible ways through which sustainable and equitable WASH services can be ensured, with a focus on the improvement of education and attainment of SDG 4. The study contributes to the debate on sustainable development and the role that WASH can play for inclusive and quality education for all, drawing from the review of best practices from the world over and policy recommendations. Also, the integration of WASH programmes into school health services supports the goals of Family Medicine and Primary Health Care by preventing diseases, improving health education and promoting healthy families and communities.

## Methods

This article relies on a narrative review of the relevant literature and adopts a literature search method. Consequently, there is a heavy reliance on secondary data. The scope of this review involved peer-reviewed journal articles, proceedings of conferences and published reports. Keywords used were ‘WASH’, ‘quality education’, ‘developing countries’, ‘strategies’, ‘challenges’, ‘prospects’ and ‘stakeholders’. Articles and reports published from the year 2015 to 2025 were utilised. Only those sources aimed at developing countries and especially those that explored the educational impact of WASH interventions were utilised. Peer-reviewed articles and grey literature were compared to represent a broad range of opinions. This study only considered publications in the English language. Several related studies^10,11,12,13^ have used this approach based on its significant advantages.

## Results and discussion

### Prospects of the implementation of WASH on the quality of education in developing countries

Water, sanitation and hygiene improvement in schools significantly adds to the education quality in developing nations. For instance, clean water and sanitation reduce the cases of waterborne diseases like cholera and typhoid, with improved student attendance and overall health. Healthy students focus well, are engaged meaningfully and perform academically.^14,15^ In addition to that, increased enrolment and retention necessitate WASH, primarily among girls. Clean, private and on-campus toilets guarantee minimal dropout rates, primarily when the girls are in their puberty stage. Private and secure latrines deter exposure to harassment, promoting gender balance.^16^

Also, there is an enhancement in learning performance and motivation along with a clean school environment. Even the educators benefit through reduced illnesses and job satisfaction, enhancing the school’s overall quality.^17,18^

Water, sanitation and hygiene interventions promote hygiene training, in which the students are taught water saving, hand washing and body washing. These habits form healthy habits that enhance health in communities.^19^

Community participation is crucial to WASH success. Planning and implementation involving communities guarantee facilities that meet student needs and guarantee sustainability. Governments, NGOs and communities come together to promote resource management and accountability.^20,21^

Water, sanitation and hygiene is a priority area for the SDGs, namely SDG 4 (quality education) and SDG 6 (clean water and sanitation). Water, sanitation and hygiene scale-up in schools enhances overall school improvement and also maintains improvement in poor conditions.^6,7^

### Challenges to the implementation of water, sanitation and hygiene programmes in developing countries

Even though WASH implementation increases the level of education, there are some problems that need to be overcome. Firstly, it is financing, as poor countries are not in a position to prioritise WASH because they lack funds. Foreign aid and public–private partnerships can assist in scaling projects.^5^ Secondly, maintenance is also a problem because of the fact that facilities need to be installed but also require continuous monitoring and maintenance.^5^ There are also cultural barriers that pose challenges as habits and knowledge regarding hygiene vary with place. There is a need for education and outreach to the community to bridge these.^22,23^ Thirdly, equity must be ensured so that WASH facilities are provided to all the students, including the disabled ones.^24^

### Key stakeholders involved in the implementation of water, sanitation and hygiene programmes in developing countries

There are several stakeholders who are accountable for WASH success. Governments, through education, water and health ministries, participate in policy formulation, funding and sanitation standards assurance.^25^ Also, local governments take charge of WASH programmes within the community. School administrators and teachers contribute to the facilitation of hygiene education, with students being extensively engaged in good practice promotion.^26^

In addition to that, technical support and finance are provided by NGOs such as WaterAid and Oxford Committee for Famine Relief (OXFAM), while governments provide finance and technical support, and grassroots communities provide labour.

Furthermore, development partners like UNICEF and the World Bank provide funding and advisory services. Also, corporate social responsibility provides funding from the private sector for infrastructure development projects and sanitary products.

Also, it involves religious institutions and community leaders in the backbone of the mobilisation efforts, whereas the role of social media is mainly to increase awareness and accountability of the stakeholders.^27^ Also, family is considered the pillar of the creation of hygiene in the home setting.^28^

Successful WASH programmes are multisectoral in nature. Sustainable WASH programmes contribute to health, improved performance at school and well-being among communities in developing and developed countries.

### Key strategies for the implementation of water, sanitation and hygiene programmes on quality education

Sustaining education in Nigeria and other developing countries like it by supporting WASH is not so much a function of sanitising buildings. Rather, it encompasses providing access to clean water and promoting sanitation with a view towards giving a better, healthier experience for learning.

Development of infrastructure is among the most important means of enhancing WASH in schools. Clean drinking water needs to be made available in schools through boreholes, rainwater harvesting facilities or agreements with water utility companies. Besides this, gender-sensitive toilets need to be built and kept in good condition for the protection of privacy and dignity, and soap-dispensing handwashing facilities need to be installed at toilets, classrooms and canteens for promoting cleanliness among the students.^29^

Another key component in the implementation of WASH involves hygiene promotion. Learning sanitation, handwashing and environmental hygiene in school settings can enable students to adopt long-term hygiene practices. In addition, conducting WASH training workshops with teachers and students enables them to appreciate the link between cleanliness, school performance and overall health.^30,31^

Community involvement is also vital for the sustainability of WASH interventions. In planning, constructing and managing WASH facilities, the parents, school committees and local leaders need to be incorporated in order to develop ownership and responsibility among them. The quality of water used within the schools should also be ensured. Periodic testing of the water and installation of systems for purification will prevent waterborne diseases among students and teachers.^5^ Schools should also encourage a cleaning culture through regular clean-up activities and proper disposal of waste; a hygienic environment translates to good health and good academic performance.^32,33^

The WASH programmes should be designed on a public–private partnership so that it can ensure sustainability. Alignment with agencies such as UNICEF, WHO and WaterAid can provide financing, technical help and equipment. Private schools can also be of assistance to WASH programmes through Corporate Social Responsibility initiatives related to the financing of the water supply networks or sanitation infrastructure.^34,35^ Adequate monitoring and evaluation will result in the desired WASH interventions. Every school should have measures in place to monitor WASH programmes for impacts on student health, attendance and hygiene habits. Student, teacher and parent opinions through periodic intervals could help identify issues in time for correction. Securing long-term financing needs at local government levels will enable rehabilitation and maintenance of WASH facilities.^36^

The other most crucial aspect is the sustainable technology of WASH intervention. Such technologies, including solar pumps, bio-toilets and eco-sanitation systems, can be utilised in schools, having small environmental impacts but long life spans.^37^

Other important components of an inclusive learning environment include gender-sensitive WASH facilities. The schools should be providing clean, private sanitation facilities that enable girls to confidently handle menstruation. The WASH intervention needs to address the needs of students with disabilities in order to promote equal access to sanitation and hygiene education.^38,39^

Advocacy and sensitisation campaigns for WASH in schools should be conducted. This can be done through media announcements, school events and meetings with the public to raise communities’ awareness of the need for WASH in education and public health. Governments should also integrate WASH into educational policy so that it becomes integrated into school planning and not an afterthought.^5,30^

By integrating WASH practice into the school system, poor nations can offer cleaner, healthier and more comfortable learning environments. This will, in turn, enhance students’ academic performance at school, reduce absenteeism and enhance overall well-being.

## Conclusion

The prospects, challenges, stakeholders and strategies for the implementation of WASH in developing countries were the topics of discussion in this paper. Four primary findings were identified. Firstly, WASH improves attendance, health, enrolment, retention, learning environment and sanitation education and is part of a larger educational advancement. Secondly, the key constraints are costs, maintenance, culture and equity and propose that investment with longer time frames with community participation is needed.

Thirdly, effective WASH must have collaboration between several sectors such as governments, NGOs, the private sector, educators, community leaders and health professionals. Fourthly, among the most crucial strategies are infrastructure development in schools, promotion of health education, community mobilisation, monitoring network of water quality, partnership and mobilisation of sustainable funding.

By incorporating WASH into education, developing nations can attain cleaner and healthier learning environments, which will ultimately improve learning outcomes and the well-being of students.

There are a number of limitations in this narrative review. Firstly, the literature search was not comprehensive, as the researcher used a small number of databases and keywords, which might have led to the exclusion of important literature, especially on the relationship between WASH and education in developing countries. Secondly, the use of only English literature might have led to language bias. As a narrative review, it does not possess the methodological rigour of systematic reviews. It was not based on a standardised protocol for study selection, data extraction and quality assessment. This makes it susceptible to bias and subjectivity, particularly with regard to the indicators of SDG 4 on school attendance and learning results. Moreover, the quality of the included studies was inconsistent, with a number being based on small sample sizes and specific intervention contexts. Inconsistency in the design and indicators of the included studies also affected the comparison. In addition, publication bias may exist, where only the positive outcomes are emphasised.

## References

[1] Pereira CT, Sorlini S, Sátiro J, Albuquerque A. Water, sanitation, and hygiene (wash) in schools: A catalyst for upholding human rights to water and sanitation in Anápolis, Brazil. Sustainability. 2024;16(13):5361. 10.3390/su16135361

[2] Setty K, Jiménez A, Willetts J, Leifels M, Bartram J. Global water, sanitation and hygiene research priorities and learning challenges under Sustainable Development Goal 6. Dev Policy Rev. 2020;38(1):64–84. 10.1111/dpr.12475 33041525 PMC7546406

[3] Deroo L, Walter E, Graham J. Monitoring and evaluation of WASH in schools programs: Lessons from implementing organizations. J Water, Sanit Hyg Dev. 2015;5(3):512–520. 10.2166/washdev.2015.026

[4] Wami M, Fisher J, Akpila S, Radu T. Current water, sanitation and hygiene (WASH) conditions in selected schools in Port Harcourt, Nigeria. Int Dev Plan Rev. 2022;44(4):435–455. 10.3828/idpr.2022.6

[5] John CK, Ajibade FO. Exploring the dynamics of WASH services: Challenges, enablers, and strategies for improvement. Discov Civ Eng. 2024;1(1):79. 10.1007/s44290-024-00085-9

[6] Hutton G, Chase C. The knowledge base for achieving the sustainable development goal targets on water supply, sanitation and hygiene. Int J Environ Res Public Health. 2016;13(6):536. 10.3390/ijerph13060536 27240389 PMC4923993

[7] Chatterley C, Slaymaker T, Badloe C, Nouvellon A, Bain R, Johnston R. Institutional WASH in the SDGs: Data gaps and opportunities for national monitoring. J Water Sanit Hyg Dev. 2018;8(4):595–606. 10.2166/washdev.2018.031

[8] Wagner JT, Pramling Samuelsson I. WASH from the START: Water, sanitation and hygiene education in preschool. Int J Early Child. 2019;51(1):5–21. 10.1007/s13158-019-00236-5

[9] Mohamed ES. Contribution of water, sanitation, hygiene and basic education to reduce under-five mortality in Sudan. J Water Sanit Hyg Dev. 2024;14(8):616–632. 10.2166/washdev.2024.215

[10] Peter NJ, Okagbue HI, Iroham CO, Opoko AP, Akinola AO. Literature review of areas of application of supply chain management in construction industry. Int J Sup Chain Mgt. 2020;9(3):273.

[11] Oloke OC, Sholanke AB, Akindele NA, Akinola AO. A short review of the concept and principles of supply chain management in building construction industry. IOP Conf Ser Earth Environ Sci. 2022;1054(1):012050. 10.1088/1755-1315/1054/1/012050

[12] Bridgette AE, Sholanke AB, Ene VO. Review of structural integrity concerns in high-rise buildings in coastal regions. Civ Eng Archit. 2024;12(5):3441–3451. 10.13189/cea.2024.120523

[13] Mushanga S, Oloke OC, Olukanni DO. A review of sustainable housing preferences and affordability. IOP Conf Ser Earth Environ Sci. 2024;1342(1):012030. 10.1088/1755-1315/1342/1/012030

[14] Afridi J, Khan F, Asimullah S, Khan J. Assessment of drinking water quality and waterborne diseases in primary schools of District Khyber, Khyber Pakhtunkhwa, Pakistan. J Res Rev. 2024;1(4):434–441.

[15] Sharma MK, Adhikari R, Khanal SP, Acharya D, Van Teijlingen E. Do school water, sanitation, and hygiene facilities affect students’ health status, attendance, and educational achievements? A qualitative study in Nepal. Health Sci Rep. 2024;7(8):e2293. 10.1002/hsr2.2293 39131595 PMC11310280

[16] Akanzum J, Pienaah CK. Review of the effects of adequate sanitary facilities on the participation and performance of the school girl child in Ghana. ISABB J Health Environ Sci. 2023;8(1):1–4. 10.5897/ISAAB-JHE2021.0073

[17] Anthonj C, Githinji S, Höser C, Stein A, Blanford J, Grossi V. Kenyan school book knowledge for water, sanitation, hygiene and health education interventions: Disconnect, integration or opportunities?. Int J Hyg Environ Health. 2021;235:113756. 10.1016/j.ijheh.2021.113756 34004452 PMC9760049

[18] Poague KI, Blanford JI, Anthonj C. Water, sanitation and hygiene in schools in low-and middle-income countries: A systematic review and implications for the COVID-19 pandemic. Int J Environ Res Public Health. 2022;19(5):3124. 10.3390/ijerph19053124 35270814 PMC8910349

[19] Wichaidit W, Steinacher R, Okal JA, et al. Effect of an equipment-behavior change intervention on handwashing behavior among primary school children in Kenya: The Povu Poa school pilot study. BMC Public Health. 2019;19(1):647. 10.1186/s12889-019-6902-2 31138168 PMC6537192

[20] Ajith V, Reshma AS, Mohan R, Ramesh MV. Empowering communities in addressing drinking water challenges using a systematic, participatory and adaptive approach and sustainable PPP model. Technol Forecast Soc Change. 2022;185:121970. 10.1016/j.techfore.2022.121970

[21] Shrestha A, Bhattarai TN, Acharya G, et al. Water, sanitation, and hygiene of Nepal: Status, challenges, and opportunities. ACS ES&T Water. 2023;3(6):1429–1453. 10.1021/acsestwater.2c00303

[22] Anderson DM, Gupta AK, Birken S, Sakas Z, Freeman MC. Successes, challenges, and support for men versus women implementers in water, sanitation, and hygiene programs: A qualitative study in rural Nepal. Int J Hyg Environ Health. 2021;236:113792. 10.1016/j.ijheh.2021.113792 34144357 PMC8325630

[23] Indarti N, Rostiani R, Megaw T, Willetts J. Women’s involvement in economic opportunities in water, sanitation and hygiene (WASH) in Indonesia: Examining personal experiences and potential for empowerment. Dev Stud Res. 2019;6(1):76–91. 10.1080/21665095.2019.1604149

[24] Adewusi AO, Oguntokun JA. Accessibility of public conveniences by students in higher education institutions. In: Akinbogun SP, Aigbavboa CO, Akinbogun OT, editors. Facility management practices: Empirical cases in developing countries. Cham: Springer Nature Switzerland, 2024; p. 81–97.

[25] Okesanya OJ, Eshun G, Ukoaka BM, et al. Water, sanitation, and hygiene (WASH) practices in Africa: Exploring the effects on public health and sustainable development plans. Trop Med Health. 2024;52(1):68. 10.1186/s41182-024-00614-3 39385262 PMC11463047

[26] Girmay AM, Weldegebriel MG, Mengesha SD, et al. Factors influencing access to basic water, sanitation, and hygiene (WASH) services in schools of Bishoftu Town, Ethiopia: A cross-sectional study. Discov Sustain. 2023;4(1):5. 10.1007/s43621-023-00122-0 PMC10618431 37920657

[27] Tsekleves E, Fonseca Braga M, Abonge C, et al. Community engagement in water, sanitation and hygiene in sub-Saharan Africa: Does it WASH?. J Water Sanit Hyg Dev. 2022;12(2):143–156. 10.2166/washdev.2022.136

[28] Abdel Wahed WY, Hefzy EM, Ahmed MI, Hamed NS. Assessment of knowledge, attitudes, and perception of health care workers regarding COVID-19, a cross-sectional study from Egypt. J Commun Health. 2020;45(6):1242–1251. 10.1007/s10900-020-00882-0 PMC7340762 32638199

[29] McMichael C. Water, sanitation and hygiene (WASH) in schools in low-income countries: A review of evidence of impact. Int J Environ Res Public Health. 2019;16(3):359. 10.3390/ijerph16030359 30696023 PMC6388361

[30] Olukanni D, Ducoste J, George T. Creating water, sanitation and hygiene (wash) program awareness in schools: A tool towards the success of community wash program. In: EDULEARN14 proceedings; Barcelona, Spain. Valencia: IATED, 2014; p. 6922–6927.

[31] Sangalang SO, Lemence AL, Ottong ZJ, et al. School water, sanitation, and hygiene (WaSH) intervention to improve malnutrition, dehydration, health literacy, and handwashing: A cluster-randomised controlled trial in Metro Manila, Philippines. BMC Public Health. 2022;22(1):2034. 10.1186/s12889-022-14398-w 36344973 PMC9641834

[32] Ahmed J, Wong LP, Chua YP, Hydrie MZ, Channa N. Drinking water, sanitation, and hygiene (WASH) situation in primary schools of Pakistan: The impact of WASH-related interventions and policy on children school performance. Environ Sci Pollut Res. 2022;29(1):1259–1277. 10.1007/s11356-021-15681-w 34355319

[33] Ngcongo MT, Tekere M. Wash and drinking water quality considerations in schools in reflection of the sustainable development goals – A review. J Water Sanit Hyg Dev. 2023;13(8):566–583. 10.2166/washdev.2023.028

[34] Rakotomanana H, Komakech JJ, Walters CN, Stoecker BJ. The WHO and UNICEF Joint Monitoring Programme (JMP) indicators for water supply, sanitation and hygiene and their association with linear growth in children 6 to 23 months in East Africa. Int J Environ Res Public Health. 2020;17(17):6262. 10.3390/ijerph17176262 32872130 PMC7503684

[35] Yates T, Zannat H, Khandaker N, Porteaud D, Bouvet F, Lantagne D. Evidence summary of water, sanitation, and hygiene (WASH) coordination in humanitarian response. Disasters. 2021;45(4):913–938. 10.1111/disa.12463 32845023

[36] McGinnis SM, McKeon T, Desai R, Ejelonu A, Laskowski S, Murphy HM. A systematic review: Costing and financing of water, sanitation, and hygiene (WASH) in schools. Int J Environ Res Public Health. 2017;14(4):442. 10.3390/ijerph14040442 28425945 PMC5409642

[37] Win CZ, Jawjit W, Thongdara R, Gheewala SH, Prapaspongsa T. Towards more sustainable Water, Sanitation and Hygiene (WASH) projects in Magway Region, Myanmar. Environ Dev Sustain. 2024;26(9):22149–22173. 10.1007/s10668-023-03727-7

[38] MacArthur J, Carrard N, Mott J, Raetz S, Siscawati M, Willetts J. Gender equality approaches in water, sanitation, and hygiene programs: Towards gender-transformative practice. Front Water. 2023;5:1090002. 10.3389/frwa.2023.1090002

[39] Robinson HJ, Barrington D, Evans B, Hutchings P, Narayanaswamy L. An analysis of gender inclusion in Water, Sanitation and Hygiene (WASH) projects: Intention vs. reality. Dev Policy Rev. 2024;42(2):e12741. 10.1111/dpr.12741

